# Effects of Blood Flow Restriction Exercise on Muscle Oxygenation and Microvascular Function in Healthy Individuals: A Narrative Review

**DOI:** 10.7759/cureus.112000

**Published:** 2026-07-03

**Authors:** Aikaterini Gakidi, Andreas Zafeiridis, Konstantina Dipla, Leonidas Kastritseas, Aikaterini Tzioutzia, Athanasios Zacharias, Georgia Pitsiou, Afroditi Boutou

**Affiliations:** 1 Department of Respiratory Failure, G. Papanikolaou Hospital, Aristotle University of Thessaloniki, Thessaloniki, GRC; 2 Laboratory of Exercise Physiology and Biochemistry, Department of Physical Education and Sports Science at Serres, Aristotle University of Thessaloniki, Serres, GRC

**Keywords:** bfr, blood flow restriction exercise, microvascular function, muscle oxygenation, near-infrared spectroscopy (nirs), reactive hyperemia

## Abstract

Blood flow restriction (BFR) training involves partial occlusion of limb blood flow during exercise, and it is known to promote gains in skeletal muscle strength and hypertrophy. However, its impact on muscle oxygenation and microvascular function remains unclear, as recent studies and reviews have mainly focused on macrovascular acute and chronic adaptations. This review aimed to examine the effects of BFR exercise on muscle oxygenation and microvascular function in healthy individuals. Research for this narrative review was conducted on PubMed/MEDLINE in January 2026. Randomized and non-randomized trials on healthy adults assessing the effects of acute or long-term BFR exercise on muscle oxygenation and microvascular function via near-infrared spectroscopy (NIRS), compared to non-BFR exercise, were included. Sixteen studies met the inclusion criteria. Acute BFR exercise was associated with significantly elevated skeletal muscle deoxygenated hemoglobin (HHb) and total hemoglobin (tHb) responses, while O_2_Hb and tissue saturation index (TSI) decreased to a greater extent compared to non-BFR exercise. Evidence on the effects of chronic BFR training on muscle oxygenation is limited but suggests a potential improvement in muscle oxygen extraction, as reflected by higher HHb levels. The few studies that examined BFR effects on microvascular function/reactive hyperemia report a greater TSI reperfusion slope during vascular occlusion tests following a BFR resistance exercise bout. BFR exercise appears to elicit greater muscle deoxygenation responses than traditional free-flow training, suggesting a stronger stimulus for chronic muscular adaptations. However, evidence regarding microvascular function/reactivity is very limited, and further research is warranted to determine the acute and chronic effects of BFR exercise.

## Introduction and background

Blood flow restriction (BFR) training, originally known as Kaatsu training, was introduced by Dr. Yoshiaki Sato over 40 years ago [1]. Exercising with BFR involves applying external pressure with a pneumatic tourniquet cuff to the most proximal region of the upper or lower limb. This pressure results in partial occlusion of blood flow to the structures distal to the cuff, almost fully restricting venous return and leading to hypoxia in the skeletal muscles. During muscle contraction, the pressure beneath the cuff increases, further restricting blood flow [2]. Studies report that BFR training with lighter external loads (20-30% one-repetition maximum, 1-RM) results in comparable muscle hypertrophy and strength to traditional heavy load resistance training (>60% 1-RM) [3,4]. Thus, this method has gained popularity as an alternative to heavy load resistance training in a variety of populations with lower physical function [4].

Given the greater hypoxic and hemodynamic stress imposed by BFR during exercise, the research interest has been directed toward its effects on skeletal muscle oxygenation and potential alterations in vascular function. Near-infrared spectroscopy (NIRS) is a non-invasive tool that is widely used to assess local tissue oxygenation and to provide a dynamic assessment of microvascular response in skeletal muscles. Specifically, NIRS measures relative changes in oxygenated (O2Hb), deoxygenated (HHb), and total (tHb) hemoglobin concentrations (μM s^−1^) and calculates tissue oxygen saturation (tissue saturation index (TSI) %) [5,6]. Changes in O_2_Hb reflect the balance in O_2_ delivery and utilization in the local tissue, HHb response is considered an index of oxygen extraction, and tHb represents changes in local blood flow [7]. Furthermore, when NIRS is applied during an occlusion-reperfusion maneuver (vascular occlusion test (VOT)), it can provide information on skeletal muscle oxidative capacity and microvascular reactivity, assessing the downstream hyperemia (reactive hyperemic response) within the skeletal muscle [6].

Most of the existing literature on vascular adaptations to BFR training has focused on macrovascular responses, suggesting improvements in flow-mediated dilatation compared to free-flow training [8-10]. Nevertheless, the effects on arterial stiffness (as assessed by cardio-ankle vascular index and ankle-brachial ratio) are conflicting [8,9]. Furthermore, a meta-analysis suggested that a BFR exercise bout elicits greater expression of endothelial function biomarkers, such as vascular endothelial growth factor (VEGF), hypoxia-inducible factor 1-alpha (HIF-1α), and endothelial nitric oxide synthase (eNOS), which may induce changes in vascular function and capillary density [11]. However, the extent to which these macrovascular adaptations and effects on endothelial function biomarkers translate to changes in microcirculatory function and skeletal muscle oxygenation kinetics is not yet clear. Therefore, this narrative review has two aims: (a) to provide a comprehensive overview of studies examining the effects of a BFR exercise bout on muscle oxygenation in healthy individuals using NIRS, and (b) to provide an overview of studies examining the vascular function at a microcirculatory level in healthy individuals, using both NIRS and VOT.

## Review

Methods

This is a narrative review, which followed the search strategy of systematic reviews, to ensure transparency and reproducibility. A literature search was conducted in January 2026 in the National Library of Medicine MEDLINE (PubMed). Combinations of the following terms: “blood flow restriction”, “blood flow restriction training”, “blood flow restriction exercise”, “blood flow occlusion”, “venous occlusion training”, “BFR”, “kaatsu training” with “endothelial function”, “near-infrared spectroscopy”, “NIRS”, “muscle”, “tissue oxygenation” and “healthy” were used in the search strategy (see Appendix 1). The inclusion criteria for the studies on healthy individuals were: (1) studies conducted on adults of any sex without any known chronic health conditions or serious cardiovascular risk and who are not professional athletes; (2) studies with at least one group using intervention based on BFR during exercise for one or more training sessions, with an applied external pressure up to 80% of limb occlusion pressure (LOP), as recommended by guidelines [2]; (3) studies that present at least one outcome related to muscle oxygenation parameter measured via NIRS during exercise (O_2_Hb, HHb, tHb, Hbdiff, TSI); (4) original studies published as full text in English in a peer-reviewed journal. Studies using whole body vibrations or any form of hypoxic training (e.g., hyperbaric chamber or high-altitude training) as the intervention group, studies only comparing baseline measures with during or post-BFR results, literature reviews, gray literature and studies that their full text of the article was unavailable, were excluded.

Results

**Figure 1:**
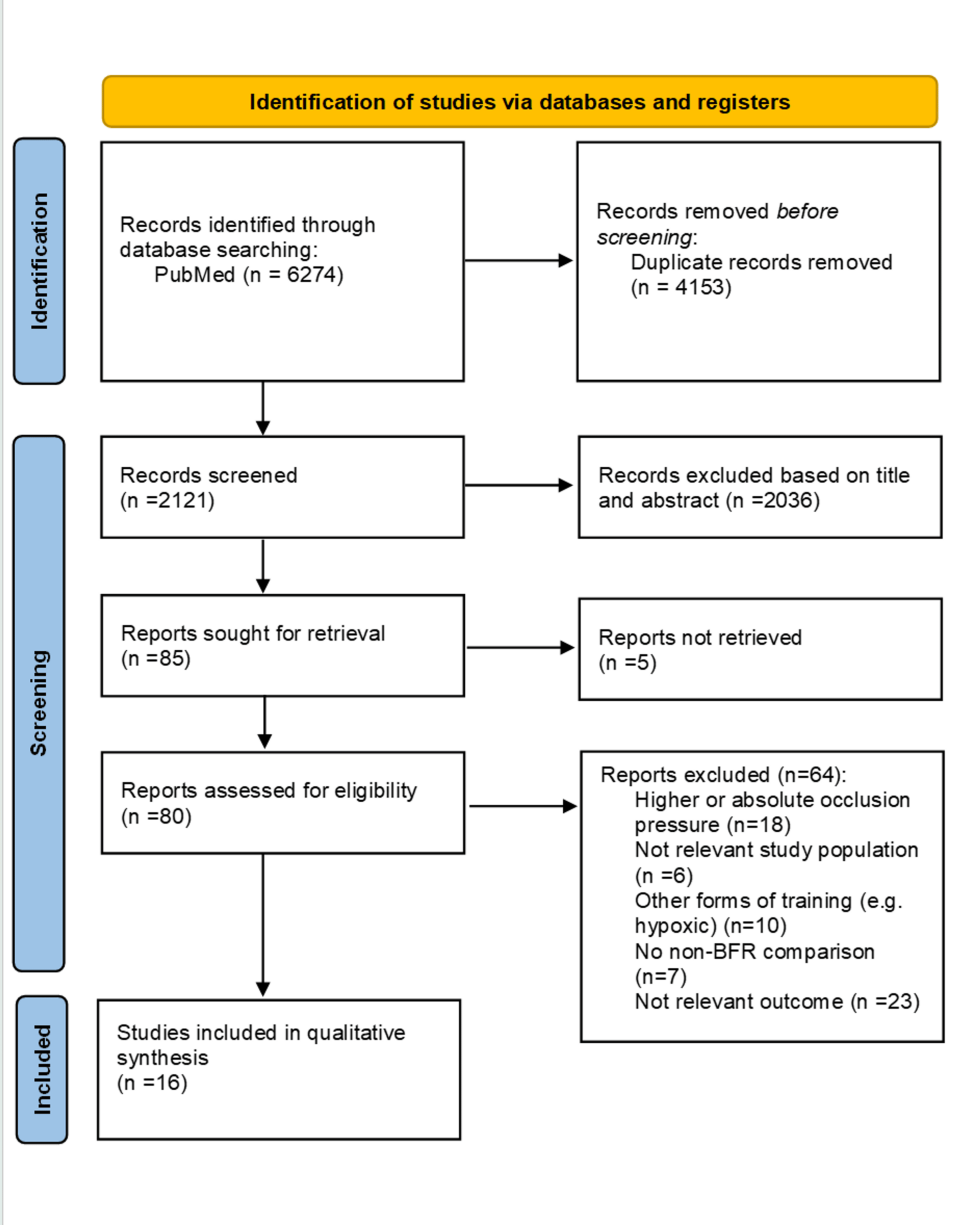
PRISMA 2020 flow diagram of the review. PRISMA: Preferred Reporting Items for Systematic Reviews and Meta-Analyses.

An initial search revealed a total of 6,274 articles. After removal of duplicates and screening titles and abstracts, 85 records were considered for full-text review. Following exclusion based on language, study design, population, and outcome relevance, 16 studies remained for qualitative synthesis (n = 205 subjects in total, 168 males and 37 females); all of thοse assessed muscle oxygenation and only one also examined microvascular function via NIRS-VOT. The Preferred Reporting Items for Systematic Reviews and Meta-Analyses (PRISMA) flow diagram of this review is presented in Figure 1. 

Regarding muscle oxygenation, all studies included young adults, and most of them were randomized controlled trials (RCTs) [12-25]. Six studies examined the acute effects of BFR resistance exercise, nine the acute effects of BFR aerobic exercise, and one study examined the effects of a medium-to-long-term training program with BFR. The studies that investigated the acute effects of BFR exercise were conducted in one to four sessions, including the control visit. NIRS measurements were mostly conducted in vastus lateralis (VL) [13,15-17,20-26], and the methodology regarding BFR application (applied pressure, cuff size, duration, and mode) was highly heterogeneous. The occlusion pressures varied from 40% of LOP [12,13,17,22,23], 45% [24], 50% [18,23], 60% [13,14,16,17,24,25,27], 70% [18] to 80% [13-15,17,19-21,26,27]. The occlusion pressure was maintained continuously in most of the studies [12-20,23,24,27], while in others the cuffs deflated during the between-sets intervals [16,22,25,26]. Regarding the cuffs’ width, two studies used small-sized cuffs 5-8 cm [12,18], 11 medium (10-13 cm) [13,14,16,17,20-24,26,27], and two studies large (15-18 cm) [19,25].

Acute Effects of Resistance Blood Flow Restriction (BFR) Exercise on Muscle Oxygenation

The resistance exercise protocols included knee extensions [13,17,25,26], barbell back squats [15], elbow flexion [12], and handgrip exercise [14], performed with BFR at low intensities (20-30% 1 RM and 20-30% maximum voluntary contraction (MVC)). The most popular exercise protocol followed Patterson et al. recommendations [2] and consisted of one set of 30 repetitions followed by three sets of 15 repetitions [13-15,17], while one study chose protocols performed until task failure [12]. In most studies, the control exercise protocol was the same as the BFR protocol but without occlusion [12-14,17,26], except for two studies that applied a higher intensity in the non-BFR (control) protocol (80-70% 1 RM) [13,15].

Oxygenated hemoglobin responses: Regarding the acute effects of BFR exercise on the changes in O_2_Hb, Garner et al. reported a significantly greater reduction in VL during low-load BFR resistance exercise at 80% LOP when compared to a free-flow work-matched protocol [26].

Deoxygenated hemoglobin responses: A consistent finding in the studies examined is the significantly higher increase in HHb during a single BFR resistance exercise bout. This is supported by studies comparing low-load resistance exercise at 60% or 80% occlusion with the same protocol without occlusion [14,17,26]. However, HHb responses were similar during low-load resistance exercise at 40% occlusion, when comparing BFR with a similar non-BFR protocol [17].

Total hemoglobin responses: The evidence regarding changes in tHb responses after applying BFR during exercise is equivocal. Garner et al. compared knee extensions at 20% 1 RM with 80% occlusion, with cuffs deflated during rest intervals, with the same protocol with free flow and found similar tHb responses in VL [26]. Reis et al. reported that during the first set of low-load knee extensions, tHB initially declined significantly with BFR (40, 60, 80% LOP) and then increased only with 40% LOP during the third set. Whereas in the non-BFR protocol, no changes in tHb were observed [17]. Finally, Perlet et al. observed a significantly greater increase in tHb during a low-load BFR barbell back squat protocol (30% 1 RM with 80% occlusion) compared to those during a traditional higher-intensity (70% 1 RM) free-flow protocol [15].

Tissue saturation index responses: Most studies suggested that TSI decreases to significantly lower levels during BFR resistance exercise compared to free flow conditions. This is supported not only by studies that compared low load resistance exercise with 40% [13,17], 60% [13,14,17], and 80% [13,14,17,26] occlusion with free flow low load resistance exercise protocols, but also by a study that compared low load BFR exercise at 40% LOP with non-BFR performed until task failure (significant differences between protocols in TSI were observed in the last five repetitions) [12]. In contrast, Ilet et al. found no differences in TSI between knee extension at 20% MVC with 40%, 60%, 80% LOP and free flow conditions [13]. When comparing BFR resistance exercise (30% 1 RM with 80% occlusion) with traditional high-intensity (70% 1 RM) non-BFR resistance exercise, Perlet et al. found a significantly greater reduction in VL during barbell back squats in the BFR protocol [15].

Upper limb exercise: Examining upper limb exercise, Ida and Sakaki suggested that the O_2_Hb and HHb responses are similar between low load elbow flexion till failure with free flow and when applying 40% occlusion during the first, middle and last five repetitions, while tHb responses are significantly higher with BFR during the first and middle five repetitions and TSI responses are significantly lower with BFR only during the last five repetitions [12]. 

Acute Effects of Aerobic Blood Flow Restriction (BFR) Exercise on Muscle Oxygenation

In studies that explored the acute effects of aerobic exercise with BFR, participants performed continuous or interval cycling bouts [16,19-21,23,24,27], running bouts on treadmill [22] or arm cranking [18]. The intensity varied across protocols, from low (25-40% VO_2 _max) to high or maximal/until exhaustion. The exercise protocols in control (non-BFR) sessions were similar to those in BFR for all studies, except for the study of Lubiak et al., where participants ran at their maximal speed without BFR and at 70%, 80%, and 90% of their peak speed in BFR protocols [22].

Oxygenated hemoglobin responses: Lavigne et al. reported similar changes in O_2_Hb during cycling at submaximal intensity between one leg that exercised at 80% LOP and the other leg exercising with free flow. In that study, the cuffs were deflated during the rest intervals [21]. In contrast, Willis et al. found a greater reduction in O_2_Hb responses during cycling bouts of 10s and 20s intervals at 20W that were repeated until exhaustion at 45% and 60% continuous occlusion on both legs, when compared to the same protocol without BFR [24].

Deoxygenated hemoglobin responses: Significantly higher increase in HHb was reported during BFR cycling (occlusions 60% and 80% LOP) at 40% VO_2_peak when compared to cycling protocols at similar (40%VO_2_peak) and higher (80%VO_2_peak) exercise intensities in non-BFR protocols [27]. In that study, the increase in HHb was also significantly higher during BFR exercise with higher occlusion pressure (80% LOP) compared to lower occlusion pressure (60% LOP) [27]. In support, Lavigne et al. also reported that the increase of HHb from rest to exercise was more pronounced for the leg that exercised with BFR during both short (60s) and long (120s) exercise intervals than for the non-occluded leg [21]. However, Willis et al. suggested that HHb increased to significantly lower levels during cycling bouts to exhaustion at 60% compared with non-BFR, and similar changes occurred when comparing 45% occlusion and free flow [24].

Total hemoglobin responses: Regarding aerobic exercise, changes in tHb during cycling were more augmented during a repeated sprint test to exhaustion with BFR [24] compared to a free-flow protocol, but were similar during similar cycling protocols at 40%WRmax between BFR and free-flow conditions [21].

Tissue saturation index responses: Examining TSI during aerobic exercise, significantly greater declines were reported in BFR compared to free flow conditions. This significantly greater response was present in studies that compared low-intensity interval cycling with 60% [16,27] and 80% [19,20,27] occlusion with free flow exercise performed either at low [16,20,27] or high intensity [19,20,27]. Kilgas et al. also reported that the TSI decline was significantly greater during 80% occlusion when compared to 60% occlusion [27]. However, recently Brown et al. found similar changes in TSI between BFR exercise with intermittent and continuous occlusion and high-intensity free-flow cycling performed in intervals [16]. Furthermore, Willis et al. compared cycling bout until exhaustion with 0%, 45%, and 60% LOP and reported a significantly greater decrease during both occlusion pressures than during free flow [24]. Finally, only two studies found a similar decrease in TSI with BFR and non-BFR [19,22]. Corvino et al. compared cycling at 30%Ppeak and 80% continuous occlusion with free flow cycling at the same intensity [19] and Lubiak et al. compared the TSI changes during the last three min of treadmill running bouts till exhaustion at 70%, 80% and 90% of the participants’ top speed with 40% occlusion with a free flow bout at 100% of their top speed [22]. Additionally, Salzmann et al. examined the effects of two BFR conditions (40% and 50% occlusion) during cycling on TSI kinetics. For the first two phases (initial drop and fast decline), there were no differences in the amplitude parameters between BFR and free flow conditions, while during the third phase (overshoot and stabilization), an increased TSI amplitude was reported with both BFR conditions compared to non-BFR [23].

Upper limb exercise: Regarding upper limb exercise, Cockfield et al. reported a greater increase in HHb responses during BFR than non-BFR exercise, as well as a significantly greater HHb increase at a higher occlusion pressure (70% LOP) when compared to lower ones (50% LOP) [18]. TSI also decreases significantly during upper limb exercise at 40% VO_2_peak with 50% and 70% occlusion when compared with non-BFR exercise at similar (40% VO_2_peak) and higher intensity (80% VO_2_peak) [18].

Effects of Training Programs With Blood Flow Restriction​​​​​​​ (BFR)​​​​​​​​​​​​​​ on Muscle Oxygenation

The study of Biazon et al. was the only one included in this review that investigated the chronic effects of a 10-week high-intensity (80% 1 RM) free flow protocol, low-load (20% 1 RM), and high-load BFR resistance training (80% 1 RM) with 60% occlusion on muscle oxygenation parameters. The participants' legs were randomly allocated to one of those three protocols. The responses of HHb were examined before and after five and 10 weeks of training. The HHb area under the curve (AUC) was greater in both high- and low-load resistance exercise with BFR after the fifth and 10th week of training, when compared to high-load free flow exercise, while no differences were present between the two BFR conditions [25].

Effects of Blood Flow Restriction​​​​​​​ (BFR)​​​​​​​​​​​​​​ Exercise on Microvascular Function

Only one study that met the inclusion criteria examined the effects of BFR on microvascular function. In the aforementioned study of Perlet et al., the participants performed barbell back squats at 30% 1 RM with 80% occlusion and at 70% 1 RM with free flow. A vascular occlusion test (VOT) was conducted pre- and post-exercise in VL and flexor carpi radialis, and TSI responses were measured during a period of complete vascular occlusion (slope 1) followed by a period of reperfusion when occlusion ended (slope 2). No significant differences were detected at pre- and post-exercise time points between BFR and non-BFR conditions in baseline TSI, minimum TSI (lowest value during occlusion), and maximum TSI (greatest value during cuff deflation) in both muscles. In VL, the rate of TSI decline in the first 30s after total occlusion (slope 1) increased to similar levels after BFR and non-BFR exercise, but the rate of TSI increase in the first 10 s of reperfusion (slope 2) was significantly greater post-exercise in the BFR condition. Interestingly, the rate of TSI increase during reperfusion of the VOT maneuver was not affected in the flexor carpi radialis muscle that served as control (neither exercise nor BFR was applied to the upper limb) [15]. Table 1 summarizes the subject characteristics as well as the intervention protocols and the outcomes of the included studies.

**Table 1:** Summary of characteristics and results of the included studies. AUC: area under the curve; BFR: blood flow restriction; FCR: flexor carpi radialis; O_2_Hb: oxygenated hemoglobin; HHb: deoxygenated hemoglobin; HI: high intensity, HL: high load; LI: low intensity, LL: Low load; LOP: limp occlusion pressure; MVC: maximum voluntary contraction; n-RCT: non-randomized controlled trial; N.R.: Not reported; Ppeak: peak power; RCT: randomized-controlled trial, RM: repetition maximum; reps: repetitions; SBP: systolic blood pressure; SD: standard deviation; tHb: total hemoglobin; TSI: tissue saturation index; VL: vastus lateralis; VO_2_peak: peak oxygen uptake; VOT: vascular occlusion test; Wmax: peak power output.

Author, year	Participants (total, male: female, age in years (mean ± SD))	Study design	Intervention	Comparison	Results
1.1. Acute effects of resistance BFR exercise on muscle oxygenation					
Garner et al., 2025 [26]	14, 14:0, 21.50 ± 2.69	n-RCT, cross-over	Knee extensions; 20% 1 RM; five sets, 15 reps, 1 min between-sets rest interval; Occlusion: 80% LOP, deflated during the rest interval; Cuff width: 11.5 cm	Same protocol, non-BFR	Muscle: VL; O_2_Hb: significantly greater decrease during BFR exercise; HHb: significantly greater increase during BFR exercise; tHb: increase in similar levels; TSI: significantly greater decrease during BFR exercise
Ilett et al., 2019 [13]	10, 10:0, 25 ± 6	RCT	Knee extensions; 20% MVC; one set of 30 reps and three sets of 15 reps, 30 sec between-sets rest interval; Occlusion: 40% (B-40), 60% (B-60), 80% (B-80) LOP, continuous; Cuff width: 10.5 cm	Non-BFR; (1) 20% MVC (LL), one set of 30 reps and three sets of 15 reps, 30 sec between-sets rest interval; (2) 80% (HL), four sets of eight reps, 2.5 min between-set interval	Muscle: VL; TSI: decrease during HL, B-40, B-60 and B-80 trials, but no change during the LL trial; TSI remained unchanged after the initial decline in all BFR trials, while recovered between sets in HL; TSI significantly lower in B-80 when compared to all other trials; TSI significantly lower in B-60 when compared to B-40, LL and HL; TSI significantly lower in B-40 when compared to LL; O_2_Hb, HHb, tHb: N.R.
Perlet et al., 2024 [15] (muscle oxygenation outcomes)	25, 14:11, 22 ± 3	RCT	Barbell back squat; 30% 1 RM; one set of 30 reps and three sets of 15 reps, 1 min between-sets rest interval; Occlusion: 80% LOP, continuous during sets and rest intervals; Cuff width: N.R.	Barbell back squat; 70% 1 RM; four sets of 10 reps 1 min between-sets rest interval; non-BFR	Muscle: VL and FCR; TSI during exercise: In FCR significantly greater decrease during non-BFR exercise, when compared to BFR and in VL significantly greater decrease during BFR exercise when compared to non-BFR; tHb: In FCR no significant differences between conditions and in VL significantly greater increase during BFR exercise; O_2_Hb, HHb: N.R.
Reis et al., 2019 [17]	13, 13:0, 23.8 ± 5.4	RCT, cross-over	Knee extensions; 20% 1 RM; one set of 30 reps and three sets of 15 reps, 30 sec between-sets rest interval for each occlusion pressure; Occlusion: 40, 60, 80% LOP, continuous pressure maintained during rest intervals; Cuff width: 13 cm	Same protocol, non-BFR	Muscle: VL; HHb: significantly greater increase during BFR exercise only with 60% and 80% occlusion; tHb: significant decrease with BFR exercise and then stable after set 1, while no changes in response to non-BFR; tHb: increased at 40% during set 3 (significantly different from non -BFR); tHb: similar endpoints at set 4; TSI: significantly greater decrease during BFR exercise; O_2_Hb: N.R.
Ida and Sakaki, 2024 [12], Experiment 1	8, 8:0, 26.9 ± 6.6	RCT	Elbow flexion; 30% 1 RM; Reps till failure; Occlusion: 40% LOP; Cuff width: 5 cm	Same protocol, non-BFR	Muscle: biceps brachii; Data were analyzed from five repetitions at the beginning (First5), in the middle (Mid5) and at the end (Last 5) of the exercise; O_2_Hb: similar changes in both conditions; HHb: similar changes in both conditions; tHb: significant increase during First5 and Mid5 with BFR; TSI: significant differences between First5 and Mid5 in both conditions; TSI: significant differences between Mid5 and Last5 in non-BFR; TSI: significant differences between conditions at Last5 (lower with BFR).
Kilgas et al., 2019 [14]	10, 10:0, 27 ± 4	RCT, cross-over	Handgrip exercise; 30% MVC; one set of 30 reps for each occlusion pressure; Occlusion: 60%, 80% LOP; Cuff width: 10 cm	One set of 30 reps, non-BFR	Muscle: flexor muscles of the forearm; HHb: significantly greater increase during exercise with 60% occlusion than with non-BFR, similar increase with 80% occlusion when compared to non-BFR; TSI: significant decrease with both 60% and 80% occlusion when compared to non-BFR; O_2_Hb, tHb: N.R.
1.2. Acute effects of aerobic BFR exercise on muscle oxygenation					
Brown et al., 2026 [16]	10, 10:0, 28.0 ± 9.0	RCT	Cycling on ergometer; 35% WRpeak; five sets of 2 min exercise, 1 min between-sets rest interval; Occlusion: 60% LOP; Cuff width: 10 cm, (1) with continuous BFR (CONT-BFR), (2) with intermittent BFR – cuffs deflated during rest intervals (INT-BFR)	Non-BFR, five sets of 2 min exercise, 1 min between-sets rest interval: (1) 35% WRpeak (LI), (2) 70% WRpeak (HI)	Muscle: VL; TSI: significantly greater decrease in INT-BFR, CONT-BFR and HI when compared to LI; TSI: no differences between INT-BFR, CONT-BFR and HI; TSI: significantly greater AUC in INT-BFR, CONT-BFR and HI when compared to LI; TSI: no differences in AUC between INT-BFR, CONT-BFR and HI; O_2_Hb, HHb, tHb: N.R.
Corvino et al., 2017 [19]	12, 12:0, 23 ± 2	RCT, cross-over	Cycling on ergometer; 30% Ppeak; two sets of five reps, each rep lasted 2 min with 1 min passive recovery between each rep and 5 min passive recovery between sets; Occlusion: 80% LOP (continuous occlusion C-BFR30); Cuff width: 18 cm	Cycling on ergometer; non-BFR; 2 sets of 5 reps, each rep lasted 2 min with 1 min passive recovery between each rep and 5 min passive recovery between sets: (1) 105% Ppeak and decrease of intensity 5% every 30 sec until the last 2 min (HI), (2) 30% Ppeak (CON30)	Muscle: gastrocnemius; TSI: similar changes between C-BFR and CON30; TSI: significantly greater decrease during C-BFR30 and CON30 when compared to HI; O_2_Hb, HHb, tHb: N.R.
Kilgas et al., 2022 [27]	10, 10:0, 26 ± 6	n-RCT	Cycling on ergometer; 40% VO_2_peak; six sets of 2 min cycling, 1 min between-sets rest interval (for each occlusion pressure); Occlusion: 60, 80% LOP, continuous; Cuff width: 10 cm	Same protocol, non-BFR at 40% VO_2_peak and 80% VO_2_peak	Muscle: rectus femoris; HHb: significantly greater increase during BFR exercise when compared to both non-BFR protocols, significantly greater increase during 80% occlusion when compared to 60% occlusion; TSI: significantly greater decrease during BFR exercise when compared to both non-BFR protocols, significantly greater decrease during 80% occlusion when compared to 60% occlusion; O_2_Hb, tHb: N.R.
Lauver et al., 2022 [20]	9, 5:4, 25 ± 5	RCT	Cycling on ergometer; five sets of 2 min exercise and 1 min rest interval: (1) 90% of WR at ventilatory threshold (90-BFR), (2) 70% of WR at ventilatory threshold (70-BFR), (3) 30% of WR at VO_2_peak (30-BFR); Occlusion: 80% LOP, continuous; Cuff width: 10 cm	Non-BFR; Cycling on ergometer; 5 sets of 2 min exercise and 1 min rest interval: (1) work intervals were performed at a work rate that would elicit a V̇O2 response corresponding to 50% of the difference between VT and V̇O2peak (HI), (2) 90% of WR at VT (CON-90)	Muscle: VL; TSI: similar changes from the baseline (decrease) in all BFR protocols; TSI: significantly greater decrease in all BFR protocols when compared to non-BFR; O_2_Hb, HHb, tHb: N.R.
Lavigne et al., 2024 [21]	8, 6:2, 23 ± 2	RCT, cross-over, split-leg	Cycling on ergometer; 40% Wmax: (1) 24 sets of 60 sec cycling and 30 sec between-sets rest interval (SI), (2) 24 sets of 120 sec cycling and 60 sec between-sets rest interval (LI); Occlusion: 80% LOP, cuff deflated during between-set intervals; Cuff width: 13 cm	One leg exercised without occlusion (non-BFR)	Muscle: VL; O_2_Hb: decrease in similar levels during LI with BFR when compared to LI non- BFR; HHb: significantly greater increase during both SI and LI with BFR when compared to SI and LI non-BFR respectively; tHb: similar increase during SI and LI with BFR when compared to SI and LI non-BFR respectively; TSI: N.R.
Lubiak et al., 2025 [22]	13, 0:13, 21.5 ± 1.0	RCT, cross-over	Treadmill running bouts; 70, 80, 90% of their top speed; 3 min for each speed, 5 min walking between maximal bouts; Occlusion: 40% LOP, applied only during running bouts; Cuff width: 10 cm	Non-BFR; Treadmill running bout; 100% of their top speed; 3 min	Muscle: VL; TSI: decrease in similar levels during the last minute of exercise; O_2_Hb, HHb, tHb: N.R.
Salzmann et al., 2021 [23]	12, 12:0, 24.3 ± 2.7	RCT, cross-over	Cycling on ergometer; 3 min of empty pedaling and 10 min at the power equivalent at 10% of the difference between the power at ventilatory threshold 1 and the maximal aerobic power; Occlusion: 40 or 50% LOP; Cuff width: 10 cm	Same protocol, non-BFR	Muscle: VL; TSI kinetics: No acceleration of the primary component and increase of phase 3 amplitude with both BFR conditions when compared to non-BFR; O_2_Hb, HHb, tHb: N.R.
Willis et al., 2018 [24]	11, 6:5, 26.7 ± 4.2	RCT, cross-over	Cycling on ergometer; Sprint bouts of 10 s and 20 s intervals of cycling at 20 W, repeated until exhaustion; Occlusion: 45, 60% LOP, continuous; Cuff width: 11 cm	Same protocol, non-BFR	Muscle: VL; O_2_Hb: significantly greater change during 60% occlusion when compared to non-BFR; HHb: increase in significantly lower levels during 60% occlusion when compared to non-BFR; HHb: similar changes during 45% occlusion and non-BFR; tHb: significantly greater increase during both 45% and 60% occlusion when compared to non-BFR; TSI: significantly greater decrease during both 45% and 60% occlusion when compared to non-BFR
Cockfield et al., 2023 [18]	10, 8:2, 25 ± 6	RCT, cross-over	Arm cranking; 40% VO_2_peak; six sets of 2 min exercise, 1 min between-sets rest interval (for each occlusion pressure); Occlusion: 50% (BFR50), 70% (BFR70) SBP, continuous; Cuff width: 5 cm	Arm cranking; six sets of 2 min exercise, 1 min between-sets rest interval (for each intensity): (1) 40% VO_2_peak (LI) and (2) 80% VO_2_peak (HI)	Muscle: triceps brachii; HHb: significantly greater increase during BFR with 70% occlusion when compared to50%; HHb: significantly greater increase during BFR with 70% and 50% occlusion when BFR was compared to non-BFR HI and LI (BFR70 > BFR50 ≈ HI > LI); TSI: significantly greater decrease during BFR exercise when compared to non-BFR HI and LI; O_2_Hb, tHb: N.R.
1.3. Effects of training programs with BFR on muscle oxygenation					
Biazon et al., 2019 [25]	30, 30:0, 22 ± 3	RCT, split-leg	Knee extensions; For 10 weeks, two days a week: (1) 80% 1 RM, three sets of 10 reps (four sets after the sixth week), 1 min between-sets rest interval or (HL-BFR), (2) 20% 1 RM three sets of 20 reps (four sets after the sixth week), 1 min between-sets rest interval (LL-BFR); Occlusion: 60% LOP, cuff deflated during rest intervals; Cuff width: 17.5 cm	High-intensity non-BFR protocol: 80% 1 RM, three sets of 10 reps (four sets after the sixth week), 1 min between-sets rest interval (HL-RT)	Muscle: VL; Assessment before the experimental protocol (T1), after 5 (T2), and 10 weeks (T3) of the experimental period; HHb: (HHb) AUC was significantly lower for all protocols in T3 compared to T2, but similar to T1; (HHb) AUC was greater during HL-BFR and LL-BFR than HL-RT, with no difference between HL-BFR and LL-BFR; O_2_Hb, tHb, TSI: N.R.
2. Effects of BFR exercise on microvascular function					
Perlet et al., 2024 [15] (microvascular function outcomes)	25, 14:11, 22 ± 3	RCT	Barbell back squat; 30% 1 RM; one set of 30 reps and three sets of 15 reps, 1 min between-sets rest interval; Occlusion: 80% LOP, continuous during sets and rest intervals; Cuff width: NR	Non-BFR; Barbell back squat; 70% 1 RM; four sets of 10 reps 1 min between-sets rest interval	Muscle: VL and FCR; VOT performed pre- and post-exercise; Baseline TSI, minimum TSI, maximum TSI (of VOT): no significant differences between BFR and non-BFR in VLR or FCR; TSI slope 1 of VOT (first 30 s after total occlusion): In FCR, non-BFR exercise increases the slope after exercise and BFR does not, but in VL increase in similar levels. of TSI slope 1; TSI slope 2 of VOT (first 10 s of reperfusion): In FCR, no significant changes in both BFR and non-BFR, but in VL significantly greater increase after BFR exercise when compared to non-BFR.

Discussion

To the authors’ knowledge, this is the first review of the effects of BFR on muscle oxygenation measured via NIRS during BFR training in healthy adults. The most consistent findings across all the different exercise protocols are the greater increase in HHb responses and the significantly greater decline in TSI when comparing BFR protocols with work-matched or higher-intensity free-flow protocols. Furthermore, O_2_Hb appears to decline more with BFR training, and tHb responses tend to be augmented.

Effects of Blood Flow Restriction​​​​​​​ (BFR)​​​​​​​​​​​​​​ Exercise on Muscle Oxygenation Parameters

Exercise is associated with an increase in HHb and a decrease in O_2_Hb [28] and a reduction in TSI [29], reflecting greater oxygen extraction and utilization by skeletal muscle compared to resting conditions. The addition of BFR during exercise partially occludes venous return, reduces blood flow, and induces turbulent arterial flow within the tissue distal to the cuff [30]. This results in local hypoxia and greater accumulation of metabolites, due to their increased production under hypoxic conditions and limited removal [30,31]. Most studies report that BFR results in lower O_2_Hb levels during exercise, indicative of a reduced oxygen availability relative to demand, increased HHb, reflecting enhanced oxygen extraction, and a greater decline in TSI% (a marker of overall muscle oxygenation). However, findings regarding O_2_Hb vary and require further investigation as O_2_Hb is affected by blood flow, while HHb is a less sensitive marker to blood volume changes [32].

Skeletal muscle tHb response is a surrogate measure of changes in tissue blood volume [33]. Elevated tHb responses during BFR exercise, shown in some studies [15,17,24], suggest elevated microvascular blood flow within the exercising muscle that appears to be influenced by the level of BFR pressure [17]. Specifically, higher BFR pressures during exercise lead to a progressive rise in tHb, likely due to venous pooling distal to the cuff [34,35]. Reis et al. suggested that levels exceeding 60% LOP do not increase tHb any further, as the responses between 60% and 80% LOP were similar [17]. Another phenomenon that may contribute to increases in tHb and warrants further investigation is the recruitment of capillaries that were not fully perfused/recruited prior to occlusion [36]. Nevertheless, blood pooling and accumulation of metabolites during BFR and reactive hyperemia when occlusion is removed lead to cellular swelling [37,38], which is associated with increased protein synthesis and contributes to muscle adaptations and growth [39-41]. The increase in tHb may also suggest enhanced microvascular vasodilation [42] and increased blood volume, which could elevate shear stress on the vascular endothelium and activate signaling pathways associated with angiogenesis and vascular remodeling [43]. The absence of significant differences in tHb reported in some studies [21,26] could be explained by the application of very high restriction pressures (80% LOP) [17,26], the deflation of cuffs during resting intervals between sets [21,26,44], or the elevated intravascular pressure generated by the skeletal muscle pump, which leads to venous outflow during low-intensity BFR exercise [34].

Acute Microvascular Responses

As the majority of previous studies investigating the effects of BFR on vascular function have focused on the macrovascular responses, the secondary aim of the review was to explore the effects on microvascular function with NIRS and VOT maneuver in healthy adults. However, only one study met the inclusion criteria. During the vascular occlusion test, Perlet et al. reported that in the vastus lateralis, the rate of TSI increase (the slope during the first 10 s of reperfusion) was faster during low-load BFR resistance exercise, compared with traditional high-load resistance exercise. The accelerated increase in TSI during reperfusion reflects improved delivery to demand and improved microvascular reactivity after BFR exercise [29]. This effect was localized to the occluded limb, as no significant differences were observed in the non-occluded limb [15]. During exercise, BFR induces local metabolite accumulation (e.g., lactate), and when the occlusion ends, local blood flow increases [45], and there is greater shear stress and increased nitric oxide (NO) release [46], leading to increased local vasodilation and increased TSI during reperfusion. However, these physiological mechanisms, although conceivable, are possible hypotheses rather than confirmed explanations, as they were not directly measured in the study.

Exercise improves microvascular function and generates a strong angiogenic signal in active muscles, resulting in a functionally important increase in capillary density and an enhancement of blood flow by the dilation of arterial vessels [43]. Local muscle hypoxia is a major contributing factor to angiogenic stimulus, leading to upregulation of HIF-1α and the subsequent transcription of VEGF [43]. Increased NO bioavailability during muscle contraction may influence HIF-1α stabilization and downstream angiogenic signaling as well [43]. The significant acute improvement in endothelial function, oxygen extraction, and microvascular reactivity after BFR exercise is probably the result of greater stimuli for microvascular adaptation due to ischemic exercise--hypoxia, elevated metabolic activity and accumulation of metabolites, shear stress, and vasodilation [15]. Acute increases in the expression of angiogenic signalling maskers, such as VEGF, HIF-1α, and eNOS mRNA, also support the hypothesis of greater levels of local hypoxia during BFR exercise [11,46].

Even though a single exercise bout can generate an adequate signal during and immediately after the session, the overall effect on tissue remodelling is only apparent as a long-term effect [43] and repeated exposure to this hypoxic and metabolically stressful environment may also mediate chronic adaptations of microvascular function. This is supported by Horiuchi et al., as an increase in the TSI AUC was present after four weeks of training. In that study, participants participated in a four-week training protocol and performed knee extensions and leg presses at 30% 1 RM with occlusion pressure at 130% of individual systolic blood pressure or at 75% 1 RM without occlusion. Microvascular function of the medial gastrocnemius was improved for both BFR and non-BFR, but greater improvements were found after BFR training, as quantified by the AUC of the TSI curve during the occlusion-reperfusion maneuver [47]. This study, however, was excluded from the review because of the high occlusion pressures applied during BFR exercise. Another result of this study was that BFR exercise also improved macrovascular function, as quantified by the decrease in heart-ankle pulse wave velocity [47]. Lavigne et al. also reported that applying BFR during exercise may improve reactive hyperemia and post-exercise recovery kinetics of muscle oxygen consumption after six weeks of training [48]. In alignment with the aforementioned, other studies have reported enhanced intramuscular oxygen delivery [49], improved filtration capacity [50,51], higher VEGF concentrations, greater capillary number, and capillary-to-muscle area ratio [52] after three to six weeks of regular BFR training. However, in those studies, the occlusion pressure levels that were applied were higher than the ones currently recommended or were not calculated as a fraction of LOP, according to guidelines.

Strengths and Limitations

The present review carries both strengths and limitations. This is the first narrative review to summarize the effects of BFR on muscle oxygenation in healthy individuals. It followed some systematic review search principles and used a transparent approach to literature identification and selection of records, including predefined eligibility criteria and up-to-date coverage of emerging evidence. This review grouped the studies based on the muscle measured, the exercise type, and acute or chronic adaptations to make the synthesis of results clearer. Despite the systematic approach, this review remains narrative in nature. Search in only one database and a lack of quality assessment of the records may introduce bias. Furthermore, the included studies exhibit limitations, such as small sample sizes, limited female representation, recruitment of young adults only, and lack of long-term intervention follow-up. Additionally, they differ in the methodology of the intervention (exercise types, intensities, duration, training loads). The application of different occlusion pressures and types of occlusions (intermittent or continuous) [53,54], as well as different cuff types [55], also affects responses. This lack of consistency poses a significant limitation in comparing the results. Even though the inclusion of diverse BFR methodologies complicates synthesis and interpretation, this was necessary as there is currently no universally accepted standard methodology. In refer to microvascular function, only one study was included and, thus, the topic is not adequately covered. Nevertheless, this lack of published data highlights the need of designing and conducting clinical trials on this research topic. Furthermore, the structured methodology and comprehensive synthesis applied in this review provide a useful foundation for future systematic reviews on muscle oxygenation and clinical studies investigating the changes in microvascular function after BFR exercise.

## Conclusions

In conclusion, this narrative review suggests that BFR training could have some clinically significant, acute effects on local muscle oxygenation in healthy adults, as reflected by changes in NIRS parameters. Increased oxygen extraction, altered local blood flow, and decreased muscle oxygen saturation are likely due to the profound hypoxic and ischemic stimuli induced by BFR. However, the effects of BFR exercise on microvascular function remain unclear and evidence is very limited, with only one study directly addressing this favorable outcome. Further research is needed in order to draw safe conclusions about the acute and long-term effects on muscle oxygenation, select the optimal BFR protocol and clarify the effects mechanisms related to vascular function.

## Appendices

Appendix 1

**Table 2:** Full search strategy. NIRS: near-infrared spectroscopy; BFR: blood flow restriction.

Combination of search terms	Database	Results retrieved
BFR AND endothelial function	PubMed/MEDLINE	54
BFR AND near-infrared spectroscopy	PubMed/MEDLINE	52
BFR AND NIRS	PubMed/MEDLINE	54
BFR AND muscle	PubMed/MEDLINE	1012
BFR AND healthy	PubMed/MEDLINE	467
BFR AND tissue oxygenation	PubMed/MEDLINE	133
blood flow restriction training AND endothelial function	PubMed/MEDLINE	117
blood flow restriction training AND near-infrared spectroscopy	PubMed/MEDLINE	49
blood flow restriction training AND NIRS	PubMed/MEDLINE	52
blood flow restriction training AND muscle	PubMed/MEDLINE	1149
blood flow restriction training AND healthy	PubMed/MEDLINE	382
blood flow restriction training AND tissue oxygenation	PubMed/MEDLINE	129
blood flow restriction exercise AND endothelial function	PubMed/MEDLINE	127
blood flow restriction exercise AND near-infrared spectroscopy	PubMed/MEDLINE	74
blood flow restriction exercise AND NIRS	PubMed/MEDLINE	79
blood flow restriction exercise AND muscle	PubMed/MEDLINE	1174
blood flow restriction exercise AND healthy	PubMed/MEDLINE	432
blood flow restriction exercise AND tissue oxygenation	PubMed/MEDLINE	173
kaatsu training AND endothelial function	PubMed/MEDLINE	5
kaatsu training AND near-infrared spectroscopy	PubMed/MEDLINE	4
kaatsu training AND NIRS	PubMed/MEDLINE	4
kaatsu training AND muscle	PubMed/MEDLINE	160
kaatsu training AND healthy	PubMed/MEDLINE	51
kaatsu training AND tissue oxygenation	PubMed/MEDLINE	11
venous occlusion training AND endothelial function	PubMed/MEDLINE	67
venous occlusion training AND near-infrared spectroscopy	PubMed/MEDLINE	7
venous occlusion training AND NIRS	PubMed/MEDLINE	7
venous occlusion training AND muscle	PubMed/MEDLINE	145
venous occlusion training AND healthy	PubMed/MEDLINE	72
venous occlusion training AND tissue oxygenation	PubMed/MEDLINE	32
